# Snowmelt Timing Regulates Community Composition, Phenology, and Physiological Performance of Alpine Plants

**DOI:** 10.3389/fpls.2018.01140

**Published:** 2018-07-31

**Authors:** Daniel E. Winkler, Ramona J. Butz, Matthew J. Germino, Keith Reinhardt, Lara M. Kueppers

**Affiliations:** ^1^School of Natural Sciences, University of California, Merced, Merced, CA, United States; ^2^United States Geological Survey, Southwest Biological Science Center, Moab, UT, United States; ^3^Department of Forestry & Wildland Resources, Humboldt State University, Arcata, CA, United States; ^4^Pacific Southwest Region, United States Department of Agriculture Forest Service, Eureka, CA, United States; ^5^United States Geological Survey, Forest and Rangeland Ecosystem Science Center, Boise, ID, United States; ^6^Department of Biological Sciences, Idaho State University, Pocatello, ID, United States; ^7^Energy & Resource Group, University of California, Berkeley, Berkeley, CA, United States

**Keywords:** distribution, elevation, flowering, Niwot Ridge, photosynthesis, snowmelt gradient, spatio-temporal dynamics, water relations

## Abstract

The spatial patterning of alpine plant communities is strongly influenced by the variation in physical factors such as temperature and moisture, which are strongly affected by snow depth and snowmelt patterns. Earlier snowmelt timing and greater soil-moisture limitations may favor wide-ranging species adapted to a broader set of ecohydrological conditions than alpine-restricted species. We asked how plant community composition, phenology, plant water relations, and photosynthetic gas exchange of alpine-restricted and wide-ranging species differ in their responses to a ca. 40-day snowmelt gradient in the Colorado Rocky Mountains (*Lewisia pygmaea*, *Sibbaldia procumbens*, and *Hymenoxys grandiflora* were alpine-restricted and *Artemisia scopulorum*, *Carex rupestris*, and *Geum rossii* were wide-ranging species). As hypothesized, species richness and foliar cover increased with earlier snowmelt, due to a greater abundance of wide-ranging species present in earlier melting plots. Flowering initiation occurred earlier with earlier snowmelt for 12 out of 19 species analyzed, while flowering duration was shortened with later snowmelt for six species (all but one were wide-ranging species). We observed >50% declines in net photosynthesis from July to September as soil moisture and plant water potentials declined. Early-season stomatal conductance was higher in wide-ranging species, indicating a more competitive strategy for water acquisition when soil moisture is high. Even so, there were no associated differences in photosynthesis or transpiration, suggesting no strong differences between these groups in physiology. Our findings reveal that plant species with different ranges (alpine-restricted vs. wide-ranging) could have differential phenological and physiological responses to snowmelt timing and associated soil moisture dry-down, and that alpine-restricted species’ performance is more sensitive to snowmelt. As a result, alpine-restricted species may serve as better indicator species than their wide-ranging heterospecifics. Overall, alpine community composition and peak % cover are strongly structured by spatio-temporal patterns in snowmelt timing. Thus, near-term, community-wide changes (or variation) in phenology and physiology in response to shifts in snowmelt timing or rates of soil dry down are likely to be contingent on the legacy of past climate on community structure.

## Introduction

Recent climate warming coupled with regional declines in winter precipitation have led to an advance in the timing of snowmelt, one of the fastest changing environmental factors in alpine systems worldwide (Dyer and Mote, 2006; Barnett et al., 2008; Clow, 2010; Stocker et al., 2013; but see Mote et al., 2018). This has potentially serious implications for alpine plant communities (Wipf et al., 2006; Wipf and Rixen, 2010) and the ecosystem services they provide (e.g., carbon sequestration, mountain resilience; Bowman and Fisk, 2001; Barni et al., 2007). Spatial variation in the timing of snowmelt generates hydroclimate gradients over very short distances, structuring alpine plant communities (e.g., moist meadows, dry meadows, snowbeds; Walker et al., 1993; Taylor and Seastedt, 1994; Choler et al., 2001; Bruun et al., 2006; Jonas et al., 2008; Litaor et al., 2008). These gradients within the alpine zone include differences in timing and duration of soil water- and nutrient-availability, pH levels, and soil organic matter content (Walker et al., 1993; Stanton et al., 1994). Hydroclimate gradients represent natural experiments that can be used to better understand ecological processes in alpine systems (e.g., Cabrera et al., 1998; Michalet et al., 2014) and can also potentially act as space-for-time substitutions (Dunne et al., 2004).

Combined with relatively short growing season lengths (<3 months), these hydroclimate and associated environmental factors result in a landscape mosaic of vegetation communities, which vary in species composition, productivity, and physiological performance (Billings and Bliss, 1959; Stanton et al., 1994; Galen and Stanton, 1995; Germino and Smith, 2001; Winkler et al., 2016a). For example, sites with earlier snowmelt are typically more productive (Kudo et al., 1999) and can have greater species richness corresponding with better soil fertility (Stanton et al., 1994). This higher richness also reflects the larger number of wide-ranging species in relatively lower elevation and/or early melting sites (Kammer and Möhl, 2002; Bruun et al., 2006; Lenoir et al., 2008; Erschbamer et al., 2009). Typically, later melting sites are where alpine-restricted or specialist species occur (Odland and Munkejord, 2008; Pickering et al., 2014). While as many as 25% of species in alpine zones can also be found below treeline (Rundel, 2011), this leaves 75% of alpine species susceptible to competition with wide-ranging species as conditions change (Bruun et al., 2006; Steinbauer et al., 2018). However, to date, no studies have compared the potentially distinct phenological and physiological sensitivities of alpine-restricted and wide-ranging species.

Changes in growing season length may negatively influence plant production and sexual reproduction indirectly via shifts in phenological cues including snowmelt timing (Kudo et al., 1999; Hülber et al., 2006; Venn and Morgan, 2007; Baptist and Choler, 2008). Individual alpine species vary in their ability to initiate growth immediately following snow retreat as a result of variation in traits like bud preformation, tolerance to chilling and photoinhibition, ability to develop under or around snow, photoperiodism, seed dormancy, and speed of development (Amen, 1965; Hamerlynck and Smith, 1994; Meloche and Diggle, 2001; Germino and Smith, 2000; Keller and Körner, 2003). Thus, snowmelt timing is expected to have varied effects on alpine plant phenology, including flowering duration; yet, we can expect that the strongest effects can be observed at early melting sites where diversity is typically higher and composed of a relatively larger number of wide-ranging species (Holway and Ward, 1963; Kudo, 1991, 1992; Kudo and Hirao, 2006; Sherwood et al., 2017). For example, Venn and Morgan (2007) found that individual species varied in their response to snowmelt timing, with some species synchronously flowering regardless of snowmelt timing due to bud preformation and others producing flowers shortly after snowmelt, thus closely tracking snowmelt. However, it remains unknown how species respond to differences in snowmelt timing as a function of their distributions.

While plant physiological performance also likely responds to snowmelt timing, this relationship has rarely been explicitly quantified as it has in phenological research (but see Oberbauer and Billings, 1981; Germino and Smith, 2001). In some temperate alpine systems, where soils rapidly dry down following snowmelt, and as environmental conditions change throughout the growing season, plants are exposed to intense radiative forcing and drier atmospheric conditions that can lead to desiccation as the season progresses (Geller and Smith, 1982; Smith and Johnson, 2009). Species–specific ecophysiological responses to snowmelt timing and soil dry-down rates may further explain patterns of alpine community composition and productivity. Adaptation to water stress and photosynthetic capacity of individual species likely contribute to individual presence in a community, with species exhibiting the highest water potentials in the wettest sites with earliest snowmelt (Oberbauer and Billings, 1981), and could be linked to whether they are alpine-restricted or wide-ranging species. Species that are wide-ranging across elevation gradients (i.e., broad environmental tolerance) may also be relatively well suited to adjust performance across small-scale gradients generated by snowmelt timing. For example, the wide-ranging *Bistorta vivipara* successfully adjusted (i.e., exhibited increased photosynthetic rates) when water was experimentally added at the end of the growing season when soils were driest (Enquist and Ebersole, 1994) but was unresponsive to watering earlier in the season when soils were likely still saturated with snowmelt runoff from higher slopes or when summer precipitation events were frequent. This may explain a similar lack of photosynthetic response observed during early- and mid-season sampling events in a similar experiment that added water above ambient precipitation levels (Bowman et al., 1995). We might further expect that alpine-restricted species are at risk of local extinction if they are not able to physiologically adjust in response to earlier snowmelt and associated exposure outside of their typical optima (Lenoir et al., 2008). Yet, whether these potential plant relationships with snowmelt gradients can be attributed to plant-soil water relationships remains an unanswered question in alpine ecology.

In this study, we utilized a ca. 40-day snowmelt gradient in the Colorado Rocky Mountains to assess the associations between snowmelt timing and alpine community composition and function. We asked how community diversity and richness varied due to snowmelt timing. We predicted, as others have shown at similar sites (Litaor et al., 2008), that community composition at this site would be tightly correlated with snowmelt patterns. We also asked how flowering phenology and plant peak % cover varied due to snowmelt date. Given the short growing season length in this and similar systems, we expected that species would initiate growth shortly after snowmelt, and develop earlier with earlier snowmelt. We expected earlier melting plots, therefore, to have longer growing seasons resulting in higher species richness and peak % cover. Finally, we asked if alpine-restricted species (*Lewisia pygmaea*, *Sibbaldia procumbens*, *Hymenoxys grandiflora*) and wide-ranging species (*Artemisia scopulorum*, *Carex rupestris*, *Geum rossii*) differed in the sensitivity of plant water potential and leaf-level gas exchange to snowmelt timing. We expected that plant water potential and gas exchange would remain high across the gradient in wide-ranging species due to these species’ presumed broad environmental tolerances. Conversely, we expected that alpine-restricted species would experience lowered water potentials and gas exchange by the end of the growing season in early melting plots reflecting greater moisture limitation compared to wide-ranging species.

## Materials and Methods

### Study Site

Our alpine research site is located at Niwot Ridge, at 3545 m in elevation in the Front Range of the Colorado Rocky Mountains (40° 03′ 14.84″ N, 105° 35′ 37.71″ W). The site is on a 15° south-southeast facing slope, 400 m above local treeline. This site is characterized by a short growing season that typically lasts from June through September (Greenland, 1989), high inter-annual variability in monthly mean temperatures and precipitation, and low growing season precipitation (Walker et al., 1995). Climate data recorded at the nearby Niwot Ridge LTER Saddle weather station show mean annual air temperature was -2.15°C and mean annual precipitation was 966 mm from 1981to 2009. Mean microclimate data were calculated across the summer growing season (1 June–30 September; **Table 1**). Approximately 80% of the precipitation falls as snow at Niwot Ridge (Blanken et al., 2009). Snow depth at the site is spatially variable and controlled by topography and westerly wind (Walker et al., 1995; Litaor et al., 2008). The soils, developed on glaciofluvial deposits or residuum derived from igneous and metamorphic rock, are mapped as Moran family and classified as lithic Cryorthents. Community composition at the site is similar to moist and dry meadow community types (May and Webber, 1982; see Winkler et al., 2016a for a detailed description of the community).

**Table 1:** Summary of microclimate data for summer 2009 (Jun 1 – Sep 30).

Snowmelt dates	*R*_solar_ (W m^-2^)	*T*_air_ (°C)	*T*_min_ (°C)	*T*_max_ (°C)	*T*_soil_ (°C)	#*T*_night_ <0°C	VPD (kPa)
149–173	441 (8)	7.9 (0.3)	3.5 (0.3)	13.4 (0.4)	6.7 (0.2)	16	0.43 (0.01)


*Microclimate data except for snowmelt dates are from an LTER climate station located nearby (<500 m away and approximately at the same elevation). Snowmelt dates indicate the plot-level range in snowmelt in Julian days in the study site. Solar radiation (R_*solar*_; 300–3000 m), air (T_*air*_), minimum air (T_*min*_), maximum air (T_*max*_), and soil (T_*soil*_) temperatures, and air vapor pressure deficit (VPD) are summer daily means. #T_*night*_ < 0°C is the number of nights during the summer when air temperatures dropped below 0°C. Numbers in parentheses are SE.*

We established 20 3 m diameter plots stratified by local elevation and aspect, as well as total plant cover. We divided plots into 4 1 m^2^ quadrants to account for any fine-scale microtopography within plots (Supplementary Figure S1). The average slope of individual plots was 16% with a range of 8.5–21.5% across plots. The first plot was snow free on May 29 and the last plot on June 22. Variation in snow depth and snowmelt timing across plots results from prevailing winds from the west, the south-easterly aspect of the site, as well as microtopography within the site.

### Field Measurements

All data were recorded during the 2009 growing season, before the plots became part of the Alpine Treeline Warming Experiment. We defined the growing season as the time from the date of snowmelt until all plants had senesced in late September. Date of snowmelt was determined by manual snow surveys carried out three times per week starting at the onset of spring snowmelt. Digital SLR planimetric photographs were taken 1.5 m above each quadrant to determine the date of snowmelt, defined as the first snow free day when all subsequent snow cover events lasted less than four continuous days.

We conducted vegetation surveys at peak community productivity (determined by weekly inspection of plant flowering phenology in each plot; Negi et al., 1992). Surveys began in the middle of July and concluded by early August following the ca. 40-day snowmelt gradient from the lowest to highest elevation plots at the site. We visually estimated peak % cover as an indirect estimate of productivity (Winkler et al., 2016a), first for the entire community and then for individual species using a 1 m^2^ survey grid divided into 10 cm^2^ units. We also estimated % cover of all other surface types (i.e., solid rock, lichen cover, bryophyte cover, bare ground, fine litter, and woody debris). We measured 45 species across our site in 2009 (Supplementary Table S1). We conducted flowering phenology surveys for all species in each quadrant weekly beginning at snowmelt and continuing until flowering ceased toward the end of the growing season. These measures were used to determine the date of first flower and flowering duration for each species in each quadrant.

We selected six species for physiological measurements during the growing season, including three alpine-restricted species that only occur in the alpine zone (*L. pygmaea*, *S. procumbens*, and *H. grandiflora*) and three wide-ranging species that occur in the alpine zone but also occur at or below treeline (*A. scopulorum*, *C. rupestris*, and *G. rossii*). We measured gas exchange in the field on intact leaves during three sampling events each lasting 2 days (early July, *n* = 19; early August, *n* = 63; and early September, *n* = 55). We randomly shuffled species and plots sampled to avoid effects of sampling time (none were detected). All measurements were conducted during the hours of peak sunlight (>90% of maximum light) using an infrared gas analyzer photosynthesis system (LI-6400XT; Li-Cor Biosciences, Lincoln, NE, United States) equipped with a 2 cm × 3 cm leaf chamber with an internal LED light source. During all measurements, chamber conditions were set to a photon flux density of 1500 μmol m^-2^ s^-1^ and CO_2_ concentration of 400 μl mol^-1^. Vapor pressure was kept at ambient levels during all measurements and temperature inside the chamber was set to match ambient air temperature using the flow of air inside the chamber. Gas exchange was calculated on a projected leaf area basis, with leaf area in the chamber determined using digital photographs of the portion of leaf area inside chamber gaskets and using image processing software (ImageJ; Scion Co., Frederick, MD, United States). During the July and September measurement events, conditions were partially cloudy; during the August measurements, conditions were sunny.

Net CO_2_ assimilation (*A*_net_), stomatal conductance (*g*), internal concentration of CO_2_ (*C*_i_), and transpiration (*E*) were calculated according to Ball (1987) and Farquhar and von Caemmerer (1982), and water-use efficiency (WUE) was calculated as *A*_net_/*E*. Following an initial measurement of *A*_net_ under saturating light, the chamber was darkened (light intensity of 0 μmol m^-2^ s^-1^), and dark respiration (*R*_d_) was recorded after CO_2_ ceased to increase in the chamber and used as an indication of growth and maintenance respiration (Atkin et al., 2000). *R*_d_ under full sun conditions can be less than in what would be measured at night (Krömer, 1995), and consequently our calculated values may be overestimated. During the third sampling event (early September), we also increased CO_2_ to 800 μmol mol^-1^ (*n* = 55) to generate information on stomatal limitation to photosynthesis (greater increases in photosynthesis with increased CO_2_ at 800 μmol mol^-1^ indicate greater stomatal limitation to photosynthesis at 400 μmol mol^-1^; Sage, 1994). One individual per species was sampled for gas exchange measurements in each plot when possible (not all species occurred in all plots). Last, we measured plant water potential on detached leaves at pre-dawn (0400–0600 h local time) using a Scholander type pressure chamber (PMS-1000; PMS Instruments, Corvallis, OR, United States). Due to the long-term nature of our research plots, pre-dawn water potential measurements were made outside of the plots on 2–3 individuals of each species at each sampling date near early-, mid- and late-melting plots.

### Statistical Analyses

We calculated alpha (average species richness per plot), beta (a measure of species turnover among plots calculated as the ratio of gamma over alpha), and gamma (total species richness across all plots) diversity, and Shannon’s *H* and Pielou’s *J* (Pielou, 1969) as measures of evenness. Diversity metrics are reported in **Table 2**. All analyses were carried out in R 3.3.2. (R Core Team, 2014). We displayed the relationships between % slope of plots, snowmelt timing, richness, and % cover across the elevation gradient of our site using the Tps command from the fields package (Nychka et al., 2015). This command uses generalized cross-validation scores to portray relationships between elevation and each variable along contours. We also used non-metric multidimensional scaling (NMS) ordination to examine associations among snowmelt date and cover variables across plots. We did the same for presence/absence of individual species, log-transforming data to account for zero values and high skew (McCune and Grace, 2002). NMS ordinations were conducted using PC-ORD (MjM Software Design; Gleneden Beach, OR, United States).

**Table 2:** Species diversity of the entire alpine plant community at our site.

Diversity measure	Value
Alpha	17
Beta	2.65
Gamma	45
Shannon’s *H*	2.10
Pielou’s *J*	0.75


**Diversity indices are defined in the “Materials and Methods” section of the text.**

Species that occurred in ten or more plots were selected for phenological analyses (*n* = 19 spp.). We used a linear mixed effects model to determine whether snowmelt timing explains date of first flower across all 19 species, with snowmelt timing as a fixed effect and plot, quadrant, and species as nested random effects to account for pseudoreplication across quadrants and species. We compared this model to a null (intercept-only model) and used the change in Akaike Information Criterion corrected for small sample sizes (ΔAICc; Johnson and Omland, 2004; Aho et al., 2014). We subsequently used linear regression to test for correlations between date of first flower and snowmelt timing, and flowering duration and snowmelt timing for individual species. We used an alpha value with Bonferroni correction (α = 0.025) to account for non-independence, but note marginally significant values (α = 0.05) when appropriate to address our increased concern over Type II error.

We used linear mixed effects models to test for differences in *A*_net_ with snowmelt timing (i.e., early, mid, and late) and group (i.e., alpine-restricted vs. wide-ranging species), their interaction, and sampling event (i.e., July, August, and September) as fixed effects. Similar to our phenology models, plot, quadrant, and species were included as nested random effects to account for pseudoreplication across quadrants and species. We compared the full model to simpler versions and used the same ΔAICc approach described above to determine the best-fit model. We carried out the same analysis for *R*_d_, *g*, *C*_i_, *E*, and WUE. When full models indicated a fixed effect was important for predicting performance, we compared least-squares means of groupings adjusted for repeated measures using the Tukey method (e.g., July vs. August sampling events, alpine-restricted vs. wide-ranging species, etc.). Last, we used the same mixed model strategy for our pre-dawn water potential measurements. Models were evaluated using the nlme and lsmeans packages (Lenth, 2016; Pinheiro et al., 2018).

## Results

### Community Responses

**FIGURE 1:**
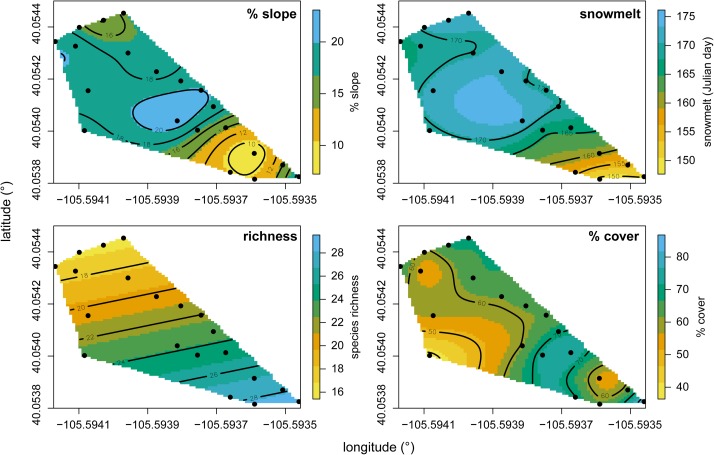
% slope, snowmelt timing (Julian day), richness, and % cover gradients fit as splines across our site. Points indicate locations of plots at our site and black contours indicate elevation. Splines were generated using generalized cross-validation scores to portray relationships between elevation and each variable along contours.

**FIGURE 2:**
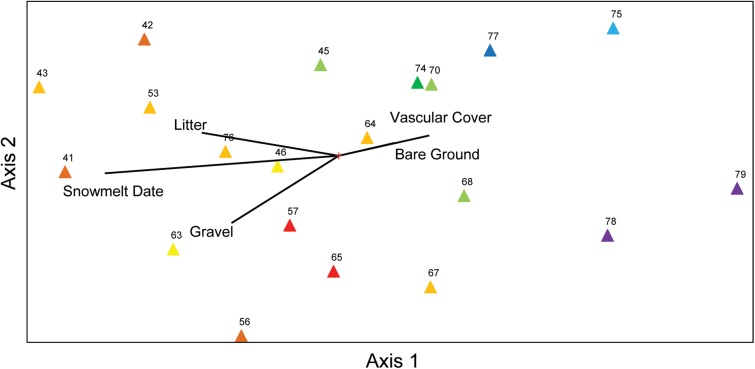
Non-metric multidimensional scaling (NMS) ordination of % cover measurements (i.e., vascular cover, bare ground, litter, gravel, and snowmelt) across the field site illustrating within community gradients. Numbers correspond to plot identifiers and colored triangles indicate snowmelt timing of each plot (warmer colors indicate later snowmelt).

**FIGURE 3:**
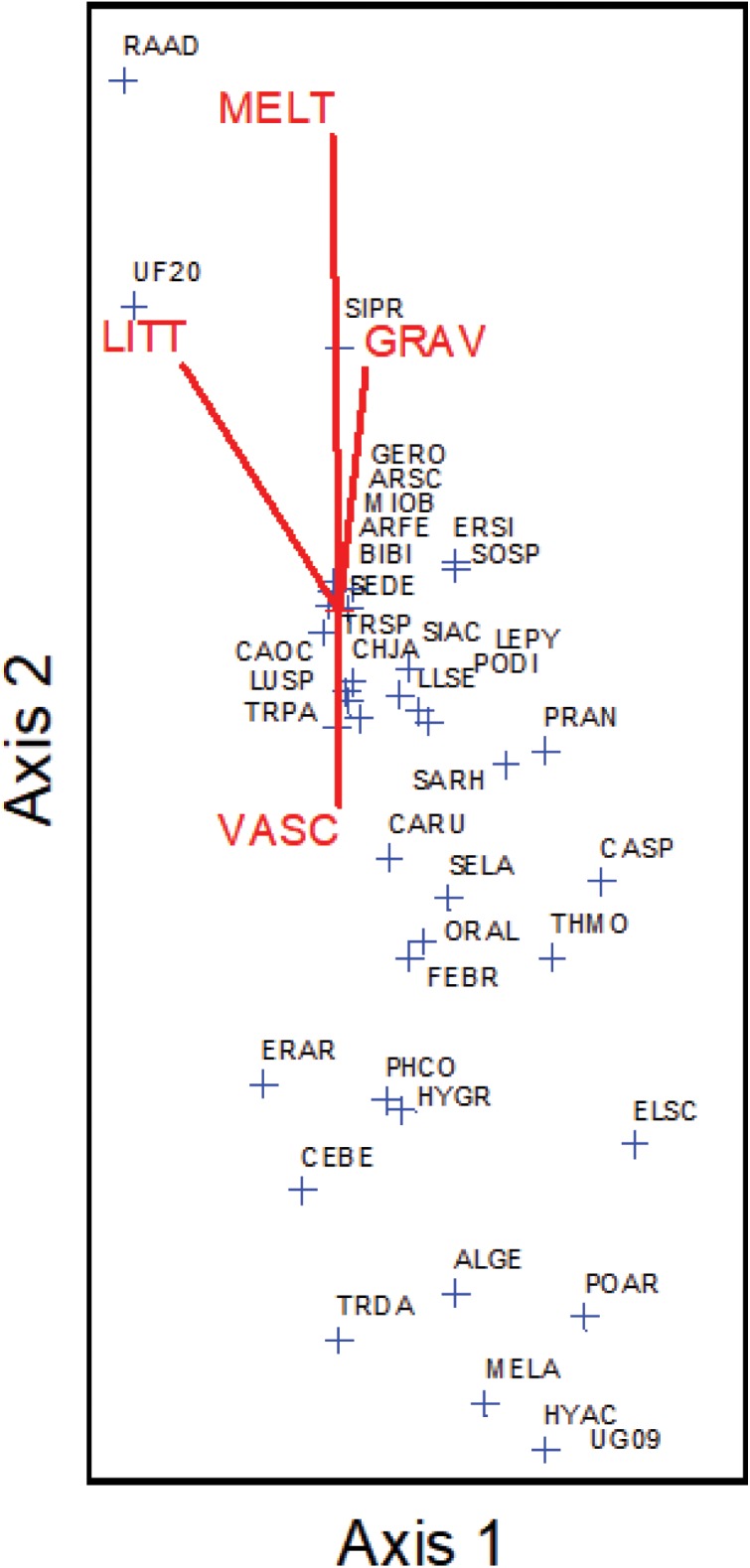
Non-metric multidimensional scaling (NMS) ordination of species presence across the site with individual species (blue crosshairs) arrayed along cover gradients (VASC = vascular plant % cover, GRAV = gravel % cover, LITT = litter % cover, and MELT = snowmelt timing). Species abbreviation codes are in Supplementary Table S1.

Species richness and total % cover by vascular plants differed in their sensitivity to elevation and snowmelt timing (**Figure 1**), with richness more closely tracking elevation and % cover more closely tracking snowmelt timing. NMS ordination of plot level % cover achieved the greatest reduction in stress with just two axes (**Figure 2**). The proportion of variance explained by the first two axes was 0.812 and 0.093 (total 0.906 explained). Vascular plant cover, litter, snowmelt timing, and gravel were significantly associated with the ordination axes. Plots with earlier snowmelt exhibited a higher percentage of vascular cover, and lower percentages of litter and gravel cover. Species compositions of plots were determined by snowmelt timing, with ca. 20% of the 45 species occurring only in the earliest or latest melting plots (**Figure 3**). A subset of ca. 25 species were found across the entire snowmelt gradient and are clustered at the center of the NMS ordination (**Figure 3**).

### Phenological Responses

Linear mixed models predicting date of first flower with snowmelt timing (AICc = 2164.77) outperformed a null model (AICc = 2196.75, ΔAICc = 31.99), suggesting that snowmelt timing has predictive power in explaining the date of first flower across our community. The same was true for the duration of flowering across our site (full model AICc = 2201.78, null model AICc = 2226.49, ΔAICc = 24.71). Of the nineteen species analyzed, twelve showed a significant positive correlation between date of first flower and snowmelt timing (*P* ≤ 0.025; with *C. rupestris* and *Trifolium parryi P* ≤ 0.05; **Table 3**), while only six species showed a significant negative correlation between flowering duration and snowmelt (*P* ≤ 0.025). Of the twelve that showed significant correlations between date of first flower and snowmelt timing, a majority of these species are wide-ranging species (*A. scopulorum, B. bistortoides, C. rupestris, Castilleja occidentalis, Erigeron simplex, G. rossii, Lloydia serotina*, and *Potentilla diversifolia, T. parryi*; **Table 3**) whereas only three are alpine-restricted species (*Chionophila jamesii*, *L. pygmaea*, and *Silene acaulis*). Of the six species that showed a significant correlation between flowering duration and snowmelt, five are wide-ranging species (*A. scopulorum, B. bistortoides, Castilleja occidentalis, E. simplex*, and *Lloydia serotina*) and only one is alpine-restricted (*L. pygmaea*; **Table 3**).

**Table 3:** Linear regressions of date of first flower (Julian day) and flowering duration (number of days) as functions of date of snowmelt (Julian day).

			First flower				Flowering duration			
				
Species		*n*	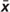 ± SEM	*r^2^*	Slope	*P*	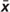 ± SEM	*r^2^*	slope	*P*
*Arenaria fendleri*	W	20	198 ± 3	0.129	0.136	0.121	41 ± 5	0.099	0.201	0.176
*Artemisia scopulorum*	W	20	193 ± 8	0.510	0.657	<0.001^∗∗∗^	24 ± 8	0.235	-0.449	0.030^∗^
*Bistorta bistortoides*	W	19	194 ± 9	0.478	0.782	0.001^∗∗∗^	35 ± 9	0.296	-0.579	0.016^∗^
*Carex rupestris*	W	16	194 ± 12	0.315	0.760	0.037^∗^	28 ± 17	0.100	-0.597	0.271
*Castilleja occidentalis*	W	14	183 ± 6	0.539	0.537	0.001^∗∗∗^	37 ± 15	0.439	-1.144	0.005^∗∗^
*Chionophila jamesii*	A	20	197 ± 8	0.326	0.574	0.009^∗∗^	20 ± 9	0.183	-0.454	0.060
*Erigeron simplex*	W	20	197 ± 6	0.766	0.670	<0.001^∗∗∗^	11 ± 6	0.394	-0.478	0.003^∗∗^
*Festuca brachyphylla*	W	15	219 ± 4	0.169	0.169	0.128	12 ± 3	0.172	-0.147	0.124
*Geum rossii*	W	20	186 ± 7	0.838	0.781	<0.001^∗∗∗^	39 ± 6	0.032	-0.142	0.449
*Lewisia pygmaea*	A	20	184 ± 7	0.604	0.678	<0.001^∗∗∗^	21 ± 8	0.269	-0.524	0.019^∗^
*Lloydia serotina*	W	16	188 ± 8	0.724	0.833	<0.001^∗∗∗^	15 ± 8	0.549	-0.668	0.001^∗∗∗^
*Luzula spicata*	W	18	217 ± 1	0.022	0.023	0.554	7 ± 3	0.001	0.015	0.890
*Minuartia obtusiloba*	A	20	189 ± 3	0.010	0.039	0.680	47 ± 8	0.072	0.264	0.252
*Potentilla diversifolia*	W	16	194 ± 7	0.411	0.473	0.007^∗∗^	26 ± 14	0.114	-0.533	0.200
*Sedum lanceolatum*	W	14	221 ± 8	0.023	0.133	0.604	14 ± 5	0.235	-0.284	0.079
*Silene acaulis*	A	10	199 ± 5	0.544	0.484	0.015^∗^	10 ± 6	0.003	0.044	0.879
*Sibbaldia procumbens*	A	12	187 ± 5	0.010	0.216	0.758	26 ± 11	0.124	1.567	0.261
*Trifolium parryi*	W	10	194 ± 7	0.414	0.539	0.045^∗^	11 ± 7	0.241	-0.404	0.150
*Trisetum spicatum*	W	20	202 ± 7	0.089	0.269	0.201	32 ± 7	0.005	-0.055	0.773


*Mean Julian day of first flower and standard error (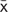
***±*** SEM) are calculated for each species across all plots it occurred in (n). (For 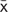, day 183 = 2 July; day 221 = 9 August). First flower: linear regression of date of first flower of each species in each plot as a function of date of snowmelt of each plot. Mean flowering duration (mean number of Julian days: 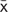) and standard deviations (***±***s) are calculated for each species across all plots it occurred in. Flowering duration: linear regression of flowering duration of each species in each plot as a function of date of snowmelt of each plot. ^∗∗∗^ ≤ 0.001, ^∗∗^ ≤ 0.01, ^∗^ ≤ 0.05. W near a species name indicates that it is a wide-ranging species. A near a species name indicates that it is an alpine-restricted species.*

### Physiological Responses

**FIGURE 4:**
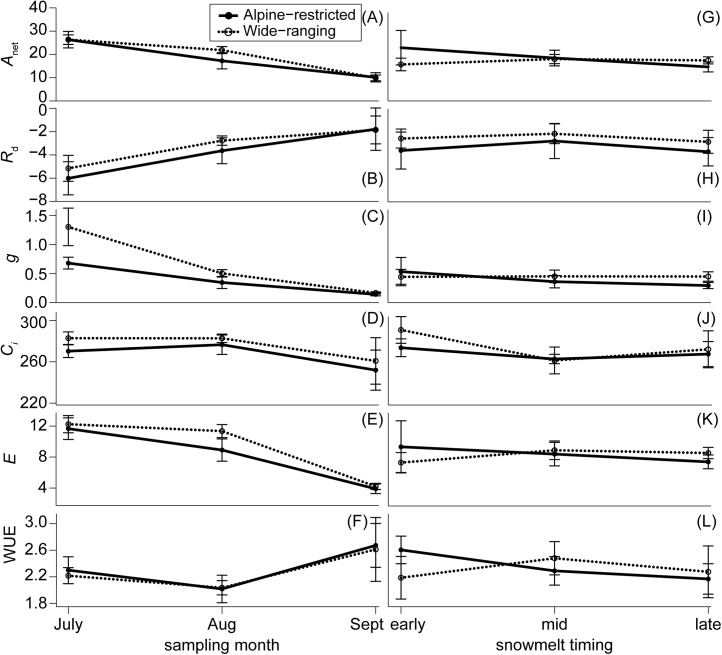
Physiological measurements separated by alpine-restricted (closed circles and solid lines) and wide-ranging species (open circles and dotted lines) by sampling month **(A–F)** and snowmelt timing **(G–L)**. Means and standard errors are presented for photosynthetic rates (*A*_net_; μmols m^-2^ s^-1^), dark respiration (*R*_d_; μmols CO_2_ m^-2^ s^-1^), stomatal conductance (*g*; mol m^-2^ s^-1^), CO_2_ assimilation (*C*_i_; PPM), transpiration (*E*; mmols H_2_O m^-2^ s^-1^), and water-use efficiency (WUE; μmol mmol^-1^).

**FIGURE 5:**
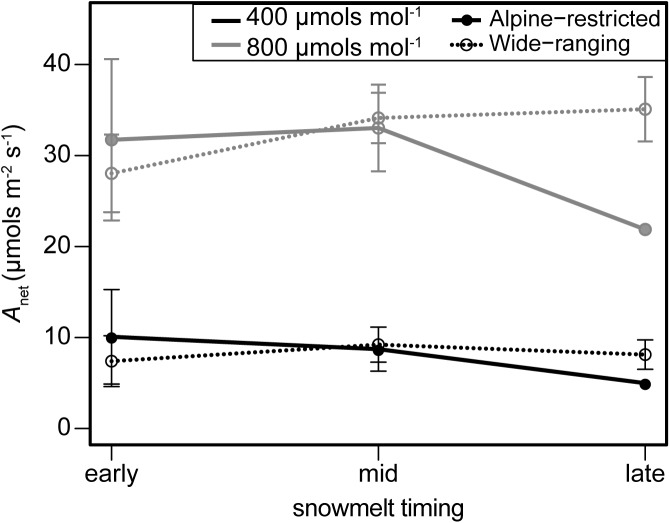
September photosynthesis (*A*_net_; μmols m^-2^ s^-1^) measured on leaves exposed to 400 μmol mol^-1^ (black lines) and 800 μmol mol^-1^ (gray lines) of CO_2_ on alpine-restricted (closed circles and soil lines) and wide-ranging species (open circles and dotted lines).

Plant gas exchange varied substantially through time but was invariant across species and the spatial snowmelt gradient (**Figure 4**). The interaction of group and snowmelt timing with sampling event as an additional covariate best predicted photosynthesis (*w*_i_ = 0.99; Supplementary Table S2). However, photosynthesis did not differ between alpine-restricted and wide-ranging species and photosynthesis declined in all species > 50% throughout the growing season (**Figure 4A**). Compared to in July, *A*_net_ was significantly lower in August (*t* = 5.41, *P* < 0.001) and September (*t* = 5.20, *P* < 0.001). Photosynthesis increased when measured at 800 μmol mol^-1^ CO_2_ compared to 400 μmol mol^-1^ CO_2_ in September (**Figure 5**), especially in wide-ranging species (though not statistically different). Increases in photosynthesis were greater in plots that had melted out late in the summer (1.05-fold increase at 800 compared to 400 μmol mol^-1^) compared to plots that had melted mid-summer (0.89-fold increase) or in early summer (0.65 increase; **Figure 5**), though these differences were not statistically significant.

Dark respiration was also best predicted by the full model with the interaction of group and snowmelt timing, with sampling event as an additional covariate (*w*_i_ = 0.70; Supplementary Table S3). Although respiration decreased by ∼50% in all species throughout the growing season, these decreases were not significantly explained by either species groupings or snowmelt timing (**Figures 4B,H**). Internal-concentration of leaf CO_2_ was also predicted best by the full model (*w*_i_ = 0.99; Supplementary Table S4), but values for the two species groups tracked each other, slightly declining throughout the season. Transpiration was also best predicted by the full model (*w*_i_ = 0.90; Supplementary Table S5). Transpiration similarly showed no difference between groups but significantly declined throughout the season; July and August values were 30–50% higher than September values (July: *t* = 4.269, *P* < 0.01; August: *t* = 6.70, *P* < 0.001; **Figures 4E,K**).

**FIGURE 6:**
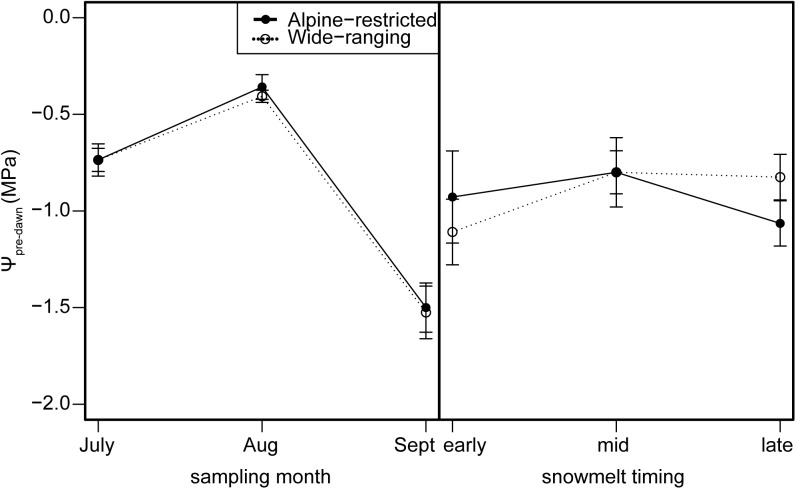
Pre-dawn leaf water potential (Ψ_pre-dawn_; MPa) measurements for alpine-restricted (closed circles and solid lines) and wide-ranging species (open circles and dotted lines) by sampling month **(left)** and snowmelt timing **(right)**.

Stomatal conductance and WUE were best explained by group and sampling event (*g*: *w*_i_ = 0.98; WUE *w*_i_ = 0.40; Supplementary Tables S6, S7). Stomatal conductance was higher for wide-ranging species than alpine-restricted species, mostly due to differences early in the summer (*t* = -2.05, *P* = 0.04; **Figures 4C,I**), though all species exhibited declines in stomatal conductance from early to late in the growing season (all *post hoc* pairwise tests had *P* < 0.001). WUE models with only snowmelt timing and sampling event performed just as strongly (*w*_i_ = 0.36, ΔAICc = 0.21; Supplementary Table S7), suggesting that sampling event is largely driving the model’s predictive power. WUE values significantly declined from July to August (*t* = -5.04, *P* < 0.001; **Figure 4F**) but there were no differences between groups, nor were there differences associated with snowmelt timing (**Figure 4L**). Pre-dawn water potential was best explained by snowmelt timing and sampling event (*w*_i_ = 0.60; Supplementary Table S8). Last, pre-dawn leaf water potential for all species increased (became less negative) by ca. 50% from July to August (*t* = -4.68, *P* < 0.001; **Figure 6**) and then significantly decreased by ca. 150% from August to September (*t* = 9.00, *P* < 0.001). However, there were no statistically significant differences due to snowmelt date (early vs. late melt: *t* = -2.69, *P* = 0.14; early vs. mid melt: *t* = -2.38, *P* = 0.19; late vs. mid melt: *t* = 0.20, *P* = 0.98; **Figure 6**).

## Discussion

Our findings reveal that spatial variation in the composition and peak % cover of this moist alpine meadow community is driven by snowmelt timing. Flowering phenology was also strongly related to snowmelt timing for a majority of species. Leaf physiology tracked seasonal soil dry-down similarly among species, regardless of whether they were alpine-restricted or wide-ranging species. While early season stomatal conductance was higher in wide-ranging species, there were no associated differences in photosynthesis or transpiration, suggesting no strong differences between these groups in physiology.

Previous research in the Mosquito Range in Colorado shows that species richness and total community cover were significantly greater in early melting microsites (Stanton et al., 1994). Our results corroborate these findings but also suggest that richness can more closely follow elevation gradients than snowmelt. Species richness and peak % cover increased with earlier snowmelt, due to a larger number of wide-ranging species present in earlier melting plots. Interestingly, peak % cover typically declined in areas where topographic depressions led to later snowmelt.

Future climate scenarios project a decrease in the snow-to-rain ratio during winter as a result of warmer temperatures (Stocker et al., 2013). Decreases in snowpack combined with warmer temperatures will ultimately lead to an advance of snowmelt and this has already been observed in montane systems (Barnett et al., 2008; Clow, 2010). Changes in snowmelt timing across a gradient or within a patch, and the associated changes in soil dry-down rates have implications for community structure and function (Pickering et al., 2014). Not only can we expect shifts in productivity, perhaps leading to a more productive alpine (Kullman, 2010; but see Baptist et al., 2010), but an overall compositional shift that will result in novel alpine communities is also possible (Alexander et al., 2015). Productivity in this system has previously been shown to be sensitive to changes in soil moisture (Winkler et al., 2016a). This was shown using a warming experiment at our site that advanced snowmelt and subsequently led to more rapid soil dry-down rates, thus influencing productivity. Our current study illustrates a similar effect of snowmelt timing but also reveals spatial structuring of the community across the gradient that contributes to productivity differences. If species are able to locally shift upward in elevation (Lenoir et al., 2008; Kiełtyk, 2017) or seek refuge in nearby microclimates (e.g., local depressions) where snowmelt changes are less pronounced (Opedal et al., 2015), productivity changes will reflect both the immediate effects of earlier snowmelt and the longer term effects of community change.

The flowering phenology of many alpine species worldwide closely tracks environmental cues including snowmelt timing, presumably as a result of pressure from relatively short growing seasons (Kudo, 1991; Walker et al., 1995; Studer et al., 2005; Kudo and Hirao, 2006; Björk and Molau, 2007). Our study reveals that flowering phenology can vary depending on whether a species’ range is restricted to the alpine or extends below treeline. We show that wide-ranging species have more flexible flowering phenologies and, in most cases, will flower longer with earlier snowmelt (nine out of twelve species with significant correlations between snowmelt timing and timing of flowering initiation were wide-ranging species) whereas alpine-restricted species appear more conservative with initiation (only 3 out of 12 species showed significant correlations with snowmelt). The same was true for duration of flowering with only one alpine-restricted species and five wide-ranging species showing a significant correlation with snowmelt. Although our results are within one community type, other studies have shown that entire communities can exhibit this relationship where more diverse communities flower earlier and more consistently (i.e., those similar to microsites where snowmelt occurs earlier in our community), while less diverse communities composed primarily of specialists (i.e., those similar to the microsites with late snowmelt in our community), are largely at the whim of snowmelt timing (Kudo and Hirao, 2006; Venn and Morgan, 2007; Baptist et al., 2010).

Many of the trends we observed in flowering phenology across the snowmelt gradient were species-level responses to snowmelt timing and related abiotic drivers. That said, these plant-environment relationships can sometimes be carried up to functional groups (Iversen et al., 2009; but see Henry and Molau, 1997). For example, nearly all of the graminoid species in our site flowered irrespective of snowmelt timing, flowering later in the season than other species on average and highlighting their late-season, drought-avoidance strategies (Rosbakh et al., 2017). However, these strategies might not always be dependent on environmental conditions experienced during the current growing season. It is estimated that ca. 50% or more of alpine plant species pre-form leaf and flower buds before the growing season begins and oftentimes 1+ years prior (Theodose et al., 1996; Körner, 2003). Since much is theorized and little is known about the prevalence of bud preformation and whether or not it occurs in many of our study species, it is difficult to say to what extent previous-years climate plays a role in the initiation of growth and flowering. However, in order to take advantage of early snowmelt, early flowering species need to maintain a high metabolic readiness while under the snow, which is dependent upon bud preformation (Körner, 1999). *G. rossii* date of first flower had the strongest correlation with date of snowmelt. *G. rossii* is not only a dominant species in our plots, but also has a long period of growth for each leaf and inflorescence (3 years) from initiation through senescence as a result of bud preformation (Meloche and Diggle, 2001). Along with temperature, the role of photoperiodism is certainly a main driver in the initiation of seasonal growth in many alpine plants (Keller and Körner, 2003). However, our data suggest that wide-ranging species may be taking advantage of growth initiation immediately after snowmelt in order to establish themselves as dominants in the community (both *G. rossii* and *C. rupestris* alternate dominance or co-dominate in our plots in terms of cover). Further studies are needed to elucidate the role of bud preformation in species at Niwot Ridge in order to concretely say what the extent of the impact of earlier snowmelt will be on these species, as well as those species whose phenologies are not correlated with snowmelt date.

Advances in snowmelt timing may provide a longer period for growth, but may also expose plants to an increased number of spring frost events that hinder growth and performance (Wipf et al., 2009; Sierra-Almeida and Cavieres, 2010). Few studies have examined alpine plant physiological connections to snowmelt timing but several studies have looked at related environmental variables that track snowmelt gradients like the one in our study (e.g., early season temperatures, species temperature optima, soil moisture status; Germino and Smith, 2000, 2001; Shen et al., 2009; Shi et al., 2010). However, our results suggest that snowmelt timing does not have a large impact on plant performance and, instead, physiological adjustment occurs in both alpine-restricted and wide-ranging species as the season progresses and environmental conditions change.

It is likely that the species in our community are displaying some level of physiological compensation by altering assimilation rates and water use along the snowmelt gradient (Ren et al., 2010). In addition to influencing composition and phenology, microclimate can also regulate plant physiological performance (Germino and Smith, 2001). We did detect higher stomatal conductance rates in wide-ranging species compared to alpine-restricted species early in the season. Even so, species overwhelmingly adjusted photosynthetic, conductance, transpiration rates, and leaf water potentials as water availability declined during the season. Water-use efficiency similarly declined from early to mid-season but increased late in the growing season when soils were driest and plants switched to investing in reproductive structures. Together, our physiological data confirm that alpine plant species are highly adapted to large amounts of environmental variation (Körner, 1999) and that responses to short-term change may not be as apparent if physiological thresholds are not crossed (Walther et al., 2002).

In this sense, the potential for species replacement or invasion increases as temperatures warm or other driving factors (e.g., soil moisture status) change with shifts in snowmelt timing. For example, snowmelt changes and subsequent increases in soil dry-down rates have already led to the encroachment of an invasive subalpine dwarf bamboo in Hokkaido, Japan (Winkler et al., 2016b). Changes in microclimates in alpine settings have the potential to negatively impact current community members by creating unfavorable conditions and creating stepping stones for non-native species to invade (Lembrechts et al., 2017). Similarly, warmer temperatures may lead to shifts in the position of local treeline (Kueppers et al., 2017), though these shifts may largely depend on available soil moisture (Moyes et al., 2013, 2015). Nonetheless, our results suggest that alpine community composition and peak % cover are strongly structured by spatio-temporal patterns in snowmelt timing, and may not change as rapidly as climate due to long generation times.

## Author Contributions

LK, MG, KR, and RB conceived and designed the experiments. RB collected the data. DW and RB analyzed the data. DW, LK, and RB wrote the manuscript. MG and KR provided editorial advice.

## Conflict of Interest Statement

The authors declare that the research was conducted in the absence of any commercial or financial relationships that could be construed as a potential conflict of interest.
