# Supplementary material for: Snowmelt Timing Regulates Community Composition, Phenology, and Physiological Performance of Alpine Plants
Source: Front Plant Sci. 2018 Jul 31;9:1140. doi: 10.3389/fpls.2018.01140 (PMC6079221; doi:10.3389/fpls.2018.01140)
Supplement: Supplementary file 1 [file Table_1.DOCX]

Supplementary Material

Snowmelt timing regulates community composition, phenology, and physiological performance of alpine plants

**[Daniel E. Winkler*,](http://loop.frontiersin.org/people/500974/overview) Ramona J. Butz, Matthew J. Germino, Keith Reinhardt, and Lara M. Kueppers**

*** Correspondence:** Corresponding Author: winklerde@gmail.com

**
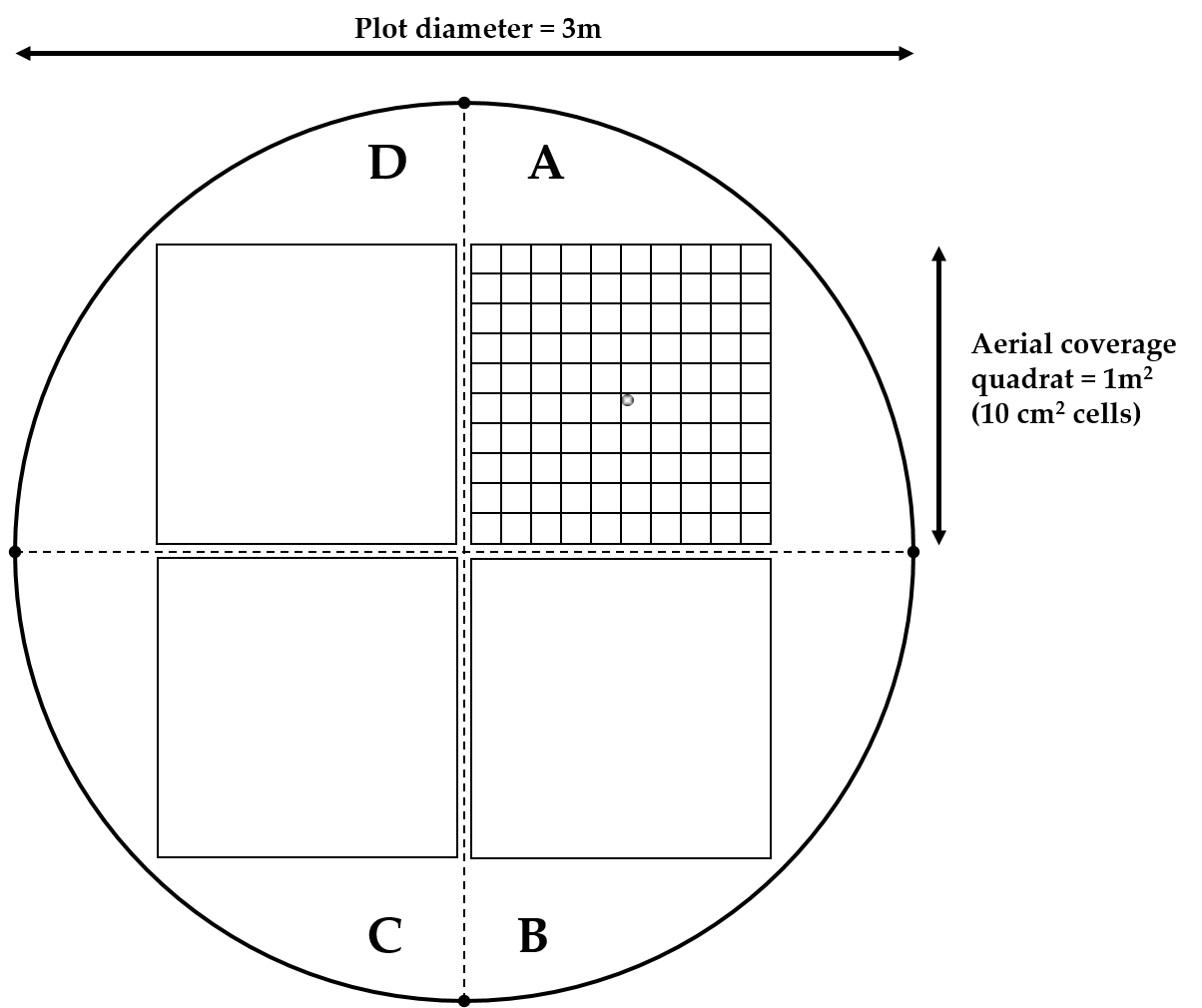
**

**Figure S1.** Plot design showing individual 1m^2^ quadrants (A–D) with an example of the 1m^2^ sampling grid divided into 10cm^2^ cells in quadrant A.

**Table S1**. List of all species measured in the plots in 2009. Not all species occurred in every plot.

| **Life form** | **Family** | **Genus** | **Species** | **Code** |
| --- | --- | --- | --- | --- |
| **Cushion/ mat-forming forb** | Caryophyllaceae | *Minuartia* | *obtusiloba* | MIOB |
|  | Caryophyllaceae | *Silene* | *acaulis* | SIAC |
|  | Fabaceae | *Trifolium* | *dasyphyllum* | TRDA |
|  | Fabaceae | *Trifolium* | *nanum* | TRNA |
|  | Polemoniaceae | *Phlox* | *condensata* | PHCO |
|  | Rosaceae | *Sibbaldia* | *procumbens* | SIPR |
| **Graminoids** | Cyperaceae | *Carex* | *rupestris* | CARU |
|  | Juncaceae | *Luzula* | *spicata* | LUSP |
|  | Poaceae | *Deschampsia* | *caespitosa* | DECA |
|  | Poaceae | *Elymus* | *scribneri* | ELSC |
|  | Poaceae | *Festuca* | *brachyphylla* | FEBR |
|  | Poaceae | *Poa* | *alpina* | POAL |
|  | Poaceae | *Poa* | *arctica* | POAR |
|  | Poaceae | *Trisetum* | *spicatum* | TRSP |
|  | Poaceae | Unknown sp. |  | UG09 |
| **Herbaceous forb** | Apiaceae | *Oreoxis* | *alpina* | ORAL |
|  | Asteraceae | *Antennaria* | *sp.* | ANTENNARIA |
|  | Asteraceae | *Artemisia* | *scopulorum* | ARSC |
|  | Asteraceae | *Erigeron* | *simplex* | ERSI |
|  | Asteraceae | *Hymenoxys* | *acaulis* | HYAC |
|  | Asteraceae | *Hymenoxys* | *grandiflora* | HYGR |
|  | Asteraceae | *Solidago* | *multiradiata* | SOMU |
|  | Boraginaceae | *Eritrichium* | *aretrioides* | ERAR |
|  | Boraginaceae | *Mertensia* | *lanceolata* | MELA |
|  | Brassicaceae | *Thlaspi* | *montanum* | THMO |
|  | Campanulaceae | *Campanula* | *rotundifolia* | CAMPANULA |
|  | Caryophyllaceae | *Arenaria* | *fendleri* | ARFE |
|  | Caryophyllaceae | *Cerastium* | *beeringianum* | CEBE |
|  | Fabaceae | *Trifolium* | *parryi* | TRPA |
|  | Liliaceae | *Allium* | *geyeri* | ALGE |
|  | Liliaceae | *Lloydia* | *serotina* | LLSE |
|  | Polygonaceae | *Bistorta* | *bistortoides* | BIBI |
|  | Ranunculaceae | *Ranunculus* | *adoneus* | RAAD |
|  | Rosaceae | *Geum* | *rossii* | GERO |
|  | Rosaceae | *Potentilla* | *diversifolia* | PODI |
|  | Saxifragaceae | *Saxifraga* | *rhomboidea* | SARH |
|  | Scrophulariaceae | *Castilleja* | *occidentalis* | CAOC |
|  | Scrophulariaceae | *Pedicularis* | *scopulorum* | PESC |
|  | Selaginellaceae | *Selaginella* | *densa* | SEDE |
|  | Unknown | Unknown sp. |  | UF20 |
| **Succulent forb** | Crassulaceae | *Sedum* | *lanceolatum* | SELA |
|  | Portulacaceae | *Lewisia* | *pygmaea* | LEPY |
|  | Primulaceae | *Primula* | *angustifolia* | PRAN |
|  | Scrophulariaceae | *Chionophila* | *jamesii* | CHJA |

**Table S2**. Results of linear mixed effects models predicting photosynthetic rates (*A_net_*) with group (alpine-restricted or wide-ranging species), snowmelt timing (melt), and sampling campaign as main effects, and plot and species as nested random effects. AICc are Akaike Information Criterion values corrected for small sample sizes. *wi* are Akaike weights, which indicate the probability of each model being the best fit relative to others shown.

| Model | AICc | ∆AICc | *k* | *w_i_* |
| --- | --- | --- | --- | --- |
| **Group * Melt + Campaign** | **1000.51** | **0.00** | **8** | **0.99** |
| Group + Melt + Campaign | 1011.18 | 10.67 | 6 | < 0.01 |
| Group + Melt | 1037.27 | 36.76 | 4 | 0 |
| Group + Campaign | 1014.27 | 13.76 | 4 | < 0.01 |
| Melt + Campaign | 1013.58 | 13.07 | 5 | < 0.01 |
| Intercept | 1043.85 | 43.34 | 1 | 0 |

**Table S3**. Results of linear mixed effects models predicting dark respiration (*R*_d_; μmols CO2 m^-2^ s^-1^) with group (alpine-restricted or wide-ranging species), snowmelt timing (melt), and sampling campaign as main effects, and plot and species as nested random effects. AICc are Akaike Information Criterion values corrected for small sample sizes. *wi* are Akaike weights, which indicate the probability of each model being the best fit relative to others shown.

| Model | AICc | ∆AICc | *k* | *w_i_* |
| --- | --- | --- | --- | --- |
| **Group * Melt + Campaign** | **843.48** | **0.00** | **8** | **0.70** |
| Group + Melt + Campaign | 847.03 | 3.55 | 6 | 0.12 |
| Group + Melt | 852.21 | 8.73 | 4 | 0.01 |
| Group + Campaign | 849.50 | 6.02 | 4 | 0.03 |
| Melt + Campaign | 846.81 | 3.33 | 5 | 0.13 |
| Intercept | 854.68 | 11.20 | 1 | 0.00 |

**Table S4**. Results of linear mixed effects models predicting CO_2_ assimilation (*C_i_*; PPM) with group (alpine-restricted or wide-ranging species), snowmelt timing (melt), and sampling campaign as main effects, and plot and species as nested random effects. AICc are Akaike Information Criterion values corrected for small sample sizes. *wi* are Akaike weights, which indicate the probability of each model being the best fit relative to others shown.

| Model | AICc | ∆AICc | *k* | *w_i_* |
| --- | --- | --- | --- | --- |
| **Group * Melt + Campaign** | **1541.79** | **0.00** | **8** | **0.99** |
| Group + Melt + Campaign | 1556.96 | 15.17 | 6 | < 0.01 |
| Group + Melt | 1569.44 | 27.65 | 4 | 0.00 |
| Group + Campaign | 1570.34 | 28.55 | 4 | 0.00 |
| Melt + Campaign | 1562.86 | 21.07 | 5 | 0.00 |
| Intercept | 1588.52 | 46.73 | 1 | 0.00 |

**Table S5**. Results of linear mixed effects models transpiration (*E*; mmols H_2_O m^-2^ s^-1^) with group (alpine-restricted or wide-ranging species), snowmelt timing (melt), and sampling campaign as main effects, and plot and species as nested random effects. AICc are Akaike Information Criterion values corrected for small sample sizes. *wi* are Akaike weights, which indicate the probability of each model being the best fit relative to others shown.

| Model | AICc | ∆AICc | *k* | *w_i_* |
| --- | --- | --- | --- | --- |
| **Group * Melt + Campaign** | **794.49** | **0.00** | **8** | **0.90** |
| Group + Melt + Campaign | 800.48 | 5.99 | 6 | 0.05 |
| Group + Melt | 830.05 | 35.56 | 4 | 0.00 |
| Group + Campaign | 800.80 | 6.31 | 4 | 0.04 |
| Melt + Campaign | 802.96 | 8.47 | 5 | 0.01 |
| Intercept | 833.82 | 39.33 | 1 | 0.00 |

**Table S6**. Results of linear mixed effects models stomatal conductance (*g*; mol m^-2^ s^-1^) with group (alpine-restricted or wide-ranging species), snowmelt timing (melt), and sampling campaign as main effects, and plot and species as nested random effects. AICc are Akaike Information Criterion values corrected for small sample sizes. *wi* are Akaike weights, which indicate the probability of each model being the best fit relative to others shown.

| Model | AICc | ∆AICc | *k* | *w_i_* |
| --- | --- | --- | --- | --- |
| Group * Melt + Campaign | 193.85 | 15.19 | 8 | < 0.01 |
| Group + Melt + Campaign | 188.34 | 9.68 | 6 | < 0.01 |
| Group + Melt | 212.23 | 33.57 | 4 | 0.00 |
| **Group + Campaign** | **178.66** | **0.00** | **4** | **0.98** |
| Melt + Campaign | 187.36 | 8.70 | 5 | 0.01 |
| Intercept | 201.42 | 22.76 | 1 | 0.00 |

**Table S7**. Results of linear mixed effects models predicting water-use efficiency (WUE; μmol mmol^-1^) with group (alpine-restricted or wide-ranging species), snowmelt timing (melt), and sampling campaign as main effects, and plot and species as nested random effects. AICc are Akaike Information Criterion values corrected for small sample sizes. *wi* are Akaike weights, which indicate the probability of each model being the best fit relative to others shown.

| Model | AICc | ∆AICc | *k* | *w_i_* |
| --- | --- | --- | --- | --- |
| Group * Melt + Campaign | 542.74 | 2.49 | 8 | 0.11 |
| Group + Melt + Campaign | 542.53 | 2.28 | 6 | 0.13 |
| Group + Melt | 553.49 | 13.24 | 4 | < 0.01 |
| **Group + Campaign** | **540.25** | **0.00** | **4** | **0.40** |
| Melt + Campaign | 540.46 | 0.21 | 5 | 0.36 |
| Intercept | 549.06 | 8.81 | 1 | < 0.01 |

**Table S8**. Results of linear mixed effects models predicting pre-dawn water potential (Ψ_pre-dawn_; MPa) with group (alpine-restricted or wide-ranging species), snowmelt timing (melt), and sampling campaign as main effects, and plot and species as nested random effects. AICc are Akaike Information Criterion values corrected for small sample sizes. *wi* are Akaike weights, which indicate the probability of each model being the best fit relative to others shown.

| Model | AICc | ∆AICc | *k* | *w_i_* |
| --- | --- | --- | --- | --- |
| Group * Melt + Campaign | 97.63 | 9.54 | 8 | < 0.01 |
| Group + Melt + Campaign | 92.69 | 4.60 | 6 | 0.06 |
| Group + Melt | 169.15 | 81.06 | 4 | 0.00 |
| Group + Campaign | 89.23 | 1.14 | 4 | 0.34 |
| **Melt + Campaign** | **88.09** | **0.00** | **5** | **0.60** |
| Intercept | 161.24 | 73.15 | 1 | 0.00 |
